# Progress in Surgery

**Published:** 1894-11-03

**Authors:** 


### PROGRESS IN SURGERY.

### SURGERY OF THE PANCREAS AND SPLEEN.

Nimier points out[1] the possibility of dealing successfully with obstruction and dilatation of the pancreatic duct by a procedure analogous to the establishment of a fistula between the dilated bile duct and the small intestine. He refers to a case recorded by Weir of cancerous obstruction of the pancreatic duct, in which it was found that there would not have been any difficulty in fixing the dilated canal to the duodenum. The chief difficulty at present is the impossibility of diagnosing with certainty obstructive dilatation of the pancreatic duct. The best way of procuring the necessary anastomosis would be by using Murphy's buttons.

**Pancreatic Cyst.**—An interesting case of one of these traumatic pancreatic effusions into the lesser peritoneal cavity is recorded[2] by J. L. Thomas. A child, aged two years, was run over across the abdomen, and three weeks afterwards had a large, tense abdominal swelling in the epigastric region. On withdrawing some of the fluid by means of a hypodermic syringe, it was found to give the usual tests for the contents of a so-called "pancreatic cyst." The cyst was emptied by aspiration, thirty-eight ounces of fluid being withdrawn. The child was known to be well eighteen months after the accident, and nothing abnormal could then be felt in his abdomen. No other case of recovery after tapping alone is recorded, and it is suggested that possibly the age of the patient in this case had something to do with the success of aspiration. As a result of experiments on the cadaver, in which the lesser peritoneal cavity was filled with fluid, and from his observation of the above case, Mr. Thomas concludes: (1) That aspiration in a young child in certain cases of traumatic effusion into the lesser peritoneal cavity is safe, and likely to be as efficacious as other methods of treatment, bearing in mind the curative effect of aspiration in empyema and other pleural effusions in infants; (2) that by careful palpation over the tumour in the cadaver the empty transverse colon could be felt, and in those rare cases in which the colon would be similarly situated, the emaciation which always obtains would materially aid in detecting it; (3) the region of the stomach should and can be avoided; (4) the space between the stomach and transverse colon is considerably increased, and with care will be pierced by the aspirating needle.

**Laparo-splenectomy for leukæmic hypertrophy** has been performed, says[3] J. P. Warbasse, twenty-eight times. Of these only one was permanently cured—a case of Franzolini's, with a moderately enlarged spleen, and only a mild leukæmia. It is therefore probable that the observations of Frazolini are faulty. From these figures it may be concluded that the leukæmic spleen is not to be extirpated, for the operation has little chance of doing any good, and in the few cases where the patient has survived the immediate effects of the operation, the disease has generally continued with increased severity. The value of the operation of splenectomy for "splenic anæmia" or simple hypertrophy has been urged by Banti. There may be no malarial history in these cases,[4] and the degree of anæmia may be slight; but the downwards course continues. Mr. Pearce Gould has performed splenectomy in a case of this class, with marked enlargement of the spleen, slight anæmia, but no leucocytosis. After operation the patient made an uninterrupted recovery, and there was a marked increase in the hæmoglobin and red blood corpuscles. W. J. Conklin after recording[5] a case of splenectomy for malarial hypertrophy, thus summarizes the present position of the operation: It is unjustifiable in leucocythæmia or other conditions in which there is extensive involvement of the lymphatic glands, or a notable increase in the white blood corpuscles. It is indicated in tumours, simple hypertrophies, and other splenic enlargements which have proved rebellious to simpler measures, and are attended with danger or serious disability. In movable or displaced spleens requiring interference extirpation is preferable to operative fixation. Severe traumatisms of the spleen, with or without an external wound, or simple prolapse of the gland into a parietal wound, demand, as a rule, immediate extirpation. In cases of protrusion experience shows that excision, partial or total, is a safer procedure than mere replacement. Removal of the spleen for cystic disease has an excellent record, but most authors advise a preliminary trial of incision and drainage. In abscess it is better, except in rare cases, to incise and drain than to attempt removal of the organ.

Dr. Tricomi reports[6] eight cases with one death. The fatal case was one of splenic leukæmia. The other cases were for wandering spleen, two; simple hyperplasia, two; and malarial hyperplasia, three. In two cases the removal of the spleen was accomplished by median laparotomy, and in the others by a curved incision extending from the lower border of the left rib to the anterior superior spine of the ilium of the same side. Prof. Terrier refers[7] to a case in which splenectomy was successfully performed for a twisted splenic pedicle. The patient had urgent symptoms of peritonitis.

**Solitary Hydatid Cysts of the Spleen.**—From a study of sixty-six cases, Trinkler[8] comes to the following conclusions: The facts cited speak clearly in favour of operative intervention in the treatment of hydatid cysts of the spleen. It is difficult to judge in theory of the merits or demerits of the various forms of laparotomy which have been recommended, but Pozzi's is the ideal method, as the cyst is removed entire. As a general rule the incision should be as long as possible. One might have to put a couple of stitches more at the top and bottom of the incision at the end of the operation, but it will give the chance to explore all parts of the tumour, to determine its boundaries, its pedicle, and, in short, one will see the actual condition of the organ, a most important factor in planning the operation.

[1] Rev. de Chir., July, 1894, and Brit. Med. Journ., Aug. 11th, 1894. [2] Lancet, March 31st, 1894, p. 799. [3] Annals of Surgery, Aug., 1894, p. 205. [4] Lancet, May 5th, 1894, p. 1,150. [5] Med. Record, July 28th, 1894, p. 103. [6] Med. Record, May 19th, 1894, p. 637. [7] Med. Week, April 20th, 1894. [8] Amer. Journ. Med. Sci., April, 1894, p. 465.

